# Limited value of current and new *in silico* predicted oocyst-specific proteins of *Toxoplasma gondii* for source-attributing serology

**DOI:** 10.3389/fpara.2023.1292322

**Published:** 2023-11-27

**Authors:** Nadia-María López-Ureña, Rafael Calero-Bernal, Bretislav Koudela, Simona Cherchi, Alessia Possenti, Fabio Tosini, Sandra Klein, Carmen San Juan-Casero, Silvia Jara-Herrera, Pikka Jokelainen, Javier Regidor-Cerrillo, Luis-Miguel Ortega-Mora, Furio Spano, Frank Seeber, Gema Álvarez-García

**Affiliations:** ^1^ Salud Veterinaria y Zoonosis (SALUVET), Animal Health Department, Veterinary Faculty, Complutense University of Madrid, Madrid, Spain; ^2^ Central European Institute of Technology (CEITEC), University of Veterinary Sciences, Brno, Czechia; ^3^ Faculty of Veterinary Medicine, University of Veterinary Sciences, Brno, Czechia; ^4^ Veterinary Research Institute, Brno, Czechia; ^5^ Unit of Foodborne and Neglected Parasitic Diseases, Department of Infectious Diseases, Istituto Superiore di Sanità, Rome, Italy; ^6^ FG16, Mycotic and Parasitic Agents and Mycobacteria, Robert Koch-Institute, Berlin, Germany; ^7^ Infectious Disease Preparedness, Statens Serum Institut, Copenhagen, Denmark; ^8^ SALUVET-Innova S.L, Veterinary Faculty, Complutense University of Madrid, Madrid, Spain

**Keywords:** *Toxoplasma gondii*, oocyst-specific proteins, diagnosis, serology, antigen prediction

## Abstract

*Toxoplasma gondii* is a zoonotic parasite infecting all warm-blooded animals, including humans. The contribution of environmental contamination by *T. gondii* oocysts to infections is understudied. The aim of the current work was to explore *T. gondii* serology as a means of attributing the source of infection using a robust stepwise approach. We identified *in silico* thirty-two promising oocyst-specific antigens from *T. gondii* ´omics data, recombinantly expressed and purified them and validated whether serology based on these proteins could discriminate oocyst- from tissue cyst-driven experimental infections. For this, three well-characterized serum panels, sampled from 0 to 6 weeks post-infection, from pigs and sheep experimentally infected with *T. gondii* oocysts or tissue cysts, were used. Candidate proteins were initially screened by Western blot with sera from pigs or sheep, infected for different times, either with oocysts or tissue cysts, as well as non-infected animals. Only the recombinant proteins TgCCp5A and TgSR1 provoked seroconversion upon infection and appeared to discriminate between oocyst- and tissue cyst-driven infections with pig sera. They were subsequently used to develop an enzyme-linked immunosorbent assay test for pigs. Based on this assay and Western blot analyses, a lack of stage specificity and low antigenicity was observed with all pig sera. The same was true for proteins TgERP, TgSporoSAG, TgOWP1 and TgOWP8, previously described as source-attributing antigens, when analyzed using the whole panels of sera. We conclude that there is currently no antigen that allows the discrimination of *T. gondii* infections acquired from either oocysts or tissue cysts by serological tests. This work provides robust new knowledge that can inform further research and development toward source-attributing *T. gondii* serology.

## 1 Introduction

*Toxoplasma gondii* (Apicomplexa) is a cosmopolitan zoonotic intracellular protist responsible for toxoplasmosis, which is considered to cause the third highest disease burden associated with food-borne infections in humans (EFSA Panel on Biological Hazards et al., 2018). Clinical toxoplasmosis usually occurs during the acute phase caused by tachyzoites (fast-replicating stage). The infection is usually mild for healthy and immunocompetent individuals, but may cause e.g. ocular disease (Gomez-Marin and de-la-Torre, 2020). However, especially immunosuppressed individuals are at risk of severe, even fatal, toxoplasmosis. When the infection occurs in pregnant women, it can lead to abortion, stillbirth, and fetal malformations. Human congenital toxoplasmosis accounts for 5.8 cases per 100,000 live births, and it is ranked among the top causes of disease burden in EU/EEA when disability-adjusted life years are considered (Cassini et al., 2018).

*T. gondii* has a complex life cycle, with a wide host range and various routes of transmission. Therefore, its control requires a One Health approach. Members of the Felidae family act as definitive hosts (DH), while almost all homeothermic animals may serve as intermediate hosts (IH) (Dubey, 2021a). All three stages of the parasite (oocyst, tachyzoite and bradyzoite) are infective to the hosts, but with differences in infectivity efficiency to IH and DH (reviewed by Dubey, 2021a). When DH ingest raw or undercooked tissues derived from IH and harboring bradyzoites-containing tissue cysts, the parasite can start both asexual and sexual multiplication in the small intestine, leading to the formation of oocysts. DH shed oocysts that are resistant to environmental stresses and that, upon sporulation, become infectious. Oocysts can contaminate soil, water bodies, vegetables, fruits and shellfish, constituting an environmental reservoir and a source of infection. While tachyzoites are usually associated with the vertical (congenital) route of transmission, bradyzoites and oocysts are responsible for most horizontal (postnatal) transmissions. Although the importance of both routes has been acknowledged (Pinto-Ferreira et al., 2019; Dubey, 2021b; López-Ureña et al., 2022), the relative importance of meat-borne *vs.* oocyst-driven transmission of *T. gondii* is still unknown. Available literature indicates that 30–60% of infections could be attributed to the meat route *vs.* 6–17% to the environmental one (Cook et al., 2000; Hald et al., 2016). A recent case-control study carried out in the Netherlands indicated a significant risk of acute toxoplasmosis through the consumption of raw or undercooked meat or meat products (Friesema et al., 2023). In contrast, in a highly endemic country such as Brazil, a compilation of outbreaks data indicated that the suspected meat-source accounted for 21.4%, whereas the environmental route was suspected in 45.2% of toxoplasmosis cases (Balbino et al., 2022). Furthermore, available data on global human toxoplasmosis outbreaks showed that 47.1% were associated with tissue cyst and 44.1% with oocyst ingestion (Pinto-Ferreira et al., 2019).

A major objective of the One Health approach is the prevention of *T. gondii* infections by designing multidisciplinary intervention strategies able to tackle the main transmission routes. Stage-specific serology could be highly useful to inform efficient interventions, and not only for humans but also for animals along the food chain. Several attempts have been made to discriminate *T. gondii* infections caused by oocysts *vs.* tissue cysts through serology. The antigens evaluated and serological assays used have been extensively reviewed (Álvarez García et al., 2021). However, validated methods are still missing. Several oocyst wall-specific proteins (TgOWP1-12; (Possenti et al., 2010; Salman et al., 2017)) and sporozoite-specific proteins (TgERP, TgSporoSAG, TgCCp5A; Radke et al., 2004; Hill et al., 2011; Santana et al., 2015) have been proposed as antigens that could serve as indicators for an oocyst-derived infection. However, contradictory results have been obtained when evaluating their potential diagnostic value. In humans, TgERP was identified as an early infection marker (Hill et al., 2011) and an indicator of environmental contamination with oocysts (Vieira et al., 2015; Mangiavacchi et al., 2016), whereas TgCCp5A was recognized by human sera in a toxoplasmosis outbreak (Santana et al., 2015). TgSporoSAG was later described as non-immunogenic in humans (Crawford et al., 2010). The assumed low level of immune stimulation by oocysts in the small intestine (Fabian et al., 2021) and the different experimental designs and procedures used by various authors indicate that it is difficult to identify antigens capable of differentiating infection routes. Accordingly, the search for such proteins should be pursued using a more standardized validation workflow (Álvarez García et al., 2021).

The present study was a comprehensive investigation of stage-specific serology aimed at identifying sporozoite- or oocyst/sporocyst wall-specific antigens. It was based on a genome-wide *in silico* prediction approach for proteins likely to be exposed to the immune system, followed by the evaluation of the derived recombinant proteins for their diagnostic potential in terms of source attribution. To this end, reference pig and sheep serum panels were selected and used in a rigorous validation workflow that consisted of screening of proteins-of-interest (POIs), development of POI-based enzyme-linked immunosorbent assay tests (ELISA) and evaluation of their analytical specificity.

## 2 Materials and methods

### 2.1 Bioinformatic analyses

#### 2.1.1 Selection of proteins-of-interest

**Figure 1:**
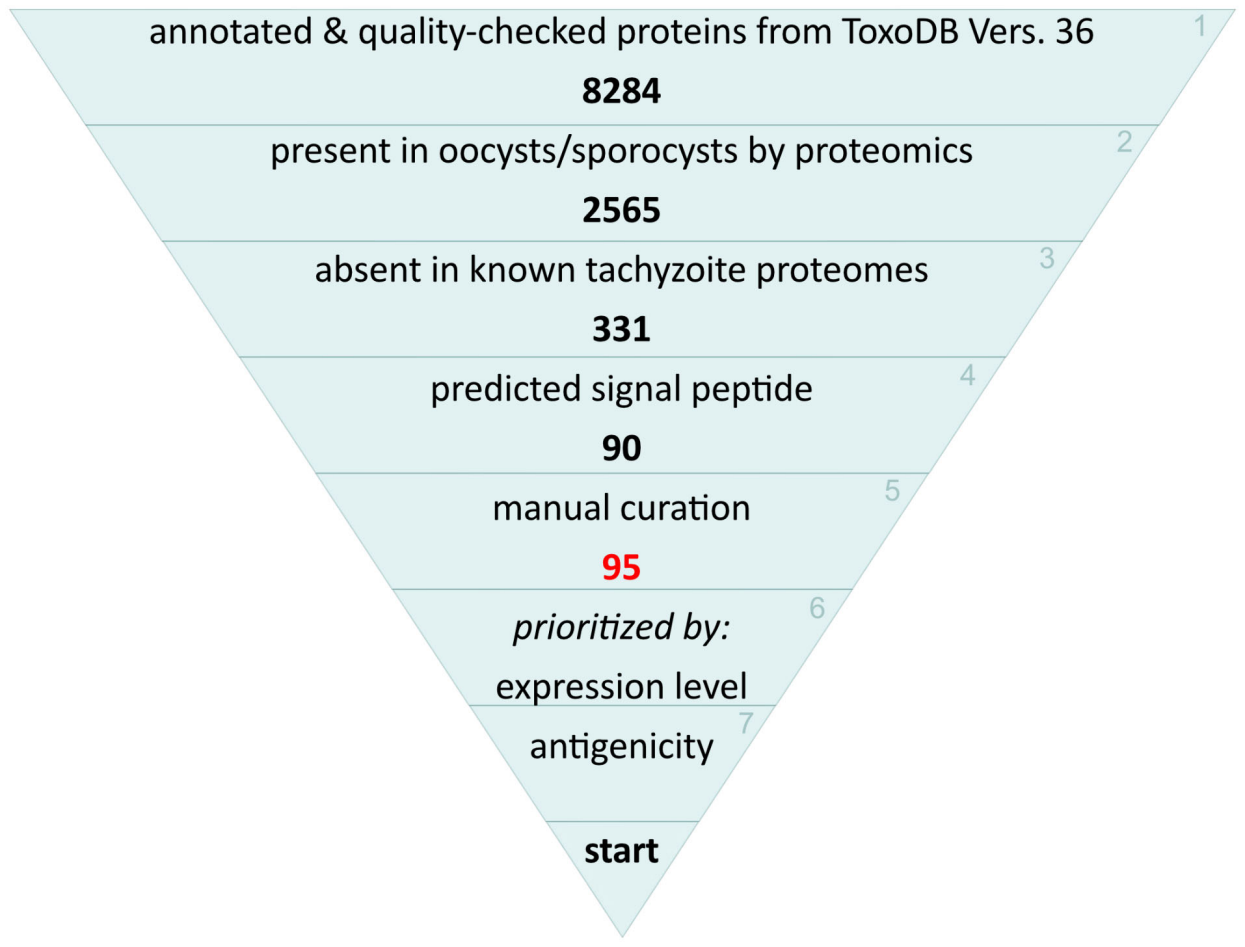
Selection and prioritizing scheme for the *in silico* prediction of *T. gondii* oocyst-derived proteins.

*T. gondii* protein candidates were identified *in silico* for oocysts-specific diagnostic purposes based on findings obtained from large-scale protein microarray approaches to identify antigens of diagnostic value from pathogens (Liang and Felgner, 2015) and taking into account the possibilities and challenges discussed previously (Álvarez García et al., 2021). Accordingly, such proteins, besides being sporozoite- or oocyst/sporocyst wall-specific, should be either secreted or surface-exposed and of relatively high abundance. Additionally, antigenicity prediction was further considered. The starting set of 8,284 annotated proteins and the algorithms and additional sources used for filtering have been previously described by us and are available as Supplementary Table 2 in Álvarez García et al. (2021) (**Figure 1**). The final 90 candidates were then manually curated for consistency and further checked based on literature data. Their designation as being Coccidia- or *T. gondii*-specific was based on Ortholog Group assignments from OrthoMCL DB (https://orthomcl.org), which can be accessed for each gene from **Supplementary Table 1**. They were verified by BLAST searches within ToxoDB (https://toxodb.org).

#### 2.1.2 Epitope predictions

We used the following algorithms for prediction of either linear or 3D epitopes, respectively, based on whole primary protein sequences: BepiPred 3 (Clifford et al., 2022); (https://services.healthtech.dtu.dk/services/BepiPred-3.0); EpiDope (Collatz et al., 2021) and DiscoTope 3 using the AlphaFold 2 mode (Høie et al., 2023); (https://services.healthtech.dtu.dk/services/DiscoTope-3.0). For seven proteins (TGME49_205658, TGME49_223700, TGME49_228240, TGME49_235200, TGME49_235315, TGME49_235390, TGME49_264070) no DiscoTope 3 predictions were performed due to the size restriction of <1,280 amino acids (aa) of AlphaFold 2/UniProt models and thus available template structures for these proteins.

As another criterion we used a representation of protein disorder either as a graphical representation of consecutive six or more disordered residues along the aa sequence of a given protein (described in Arranz-Solís et al., 2023), based on the consensus disorder predictions from MobiDB (Piovesan et al., 2020), or the pLDDT score provided by DiscoTope 3, a per-residue measure of AlphaFold2’s model accuracy. A score of <0.5 can be regarded as a strong predictor of disorder (Jumper et al., 2021). Disordered regions of proteins can be fairly antigenic (MacRaild et al., 2016; Uversky and Van Regenmortel, 2021), also reflected e.g. in some TgGRA proteins in **Supplementary Figure 1**. The algorithm-specific values for each individual aa were combined into a single graph for each protein and represented the individual scores along the protein sequence. For BepiPred 3 and EpiDope, the default thresholds of 0.15 and 0.82, respectively, are indicated. No threshold exists for DiscoTope 3 (but we used 0.25 for calculation of the combined epidope prediction score (cEPS)), instead, higher values are better, but should be considered together with the quality of the AlphaFold2 model. For calculation of cEPS see **Supplementary Figure 1B**.

### 2.2 Cloning of selected protein candidates

*T. gondii* nucleotide sequences encoding selected full-length proteins or portions thereof were amplified by PCR using as template either the genomic DNA of strain ME49 (type II) or a lambdaTriplex cDNA library from partially sporulated oocysts (strain VEG, type III), kindly provided by Dr. M. White (Montana State University, MT, USA). Leader peptides, as predicted by SignalP 5.0, (https://services.healthtech.dtu.dk/services/SignalP-5.0/) were always excluded from the amplified DNA regions. Amplicons were either cloned via Gibson assembly (NEB) in frame with the N-terminal six histidine tag (6His tag) encoded by the pQE80L (Qiagen) expression vector, linearized with restriction enzymes *Bam*HI and *Hin*dIII, or ligated at the appropriate restriction sites of plasmid pQE30 in frame with the N-terminal 6His tag. Gene-of-interest (GOI)-specific primers (**Supplementary Table 2**) consisted of a 22-28 nt sequence complementary to the open reading frame of interest preceded by the respective sequences complementary to the ends of the linearized plasmid. Alternatively, for cloning into pAviTag-C-Kan (Lucigen) as fusion with a C-terminal 6His tag, we first generated pAviTag-ccdB. It contains the counterselectable ccdB toxin amplified from pDONR221 (Invitrogen), which was inserted into pAviTag-C-Kan according to the manufacturer’s protocol, using primers rki1 and rki2. For cloning of the GOIs, pAviTag-ccdB was linearized with *Sap*I, thereby releasing the ccdB insert, and the amplified GOI open reading frames were inserted via Gibson assembly using the respective primers given in **Supplementary Table 2**. A similar approach was followed to clone GOIs as N-terminal fusions with MBP and C-terminal 6His tag. pAviTag-MBP-SAG1 (Klein et al., 2020), cut with *Bam*HI and *Pst*I to release the SAG1 insert, was used as template for the insertion of ccdB via homologous recombination using primers rki3 and rki4. This resulted in pAvi-MBP-ccdB. For in-frame cloning of GOIs into this plasmid, we amplified it using primers rki5 and rki6 (thereby releasing ccdB) and combined the amplicon with the genes amplified with the respective GOI-specific primers (**Supplementary Table 2**) using Gibson assembly. The correct insertion of the amplicons in all assembled expression vectors was verified by sequencing.

### 2.3 Expression and purification of proteins-of-interest

Consistent with the prediction rate of several algorithms (fDETECT; Meng et al., 2017, or Protein-Sol; Hebditch et al., 2017), that indicated low solubility for more than half of the 95 selected proteins (data not shown), only 32 proteins could be sufficiently purified. The pQE80L encoded proteins were expressed in the *Escherichia coli* strain DH5alpha for 3 hrs at 37 °C upon addition of 1 mM isopropyl-β-D-1-galactopyranoside. The histidine-tagged polypeptides were purified from total bacterial lysates by nickel affinity chromatography under denaturing conditions (8 M urea, 0.1 M sodium phosphate, 0.01 M Tris, pH 8.0) and successively dialyzed against multiple changes of PBS, pH 7.2. The concentration of the recombinant proteins was determined by the Bradford method (Bio-Rad). For pAviTag-based proteins, *E. coli* T7 Shuffle (NEB) was used as an expression host. Their induction and subsequent purification were done as described previously (Klein et al., 2020). For all purified proteins, their integrity and identity were subsequently determined by SDS-PAGE and Western blot (WB) analysis using anti-6His antibodies (Qiagen). They usually reached a purity of 90-95% as judged by SDS-PAGE (data not shown).

### 2.4 Experimental design for proteins-of interest screening and ELISA development

**Figure 2:**
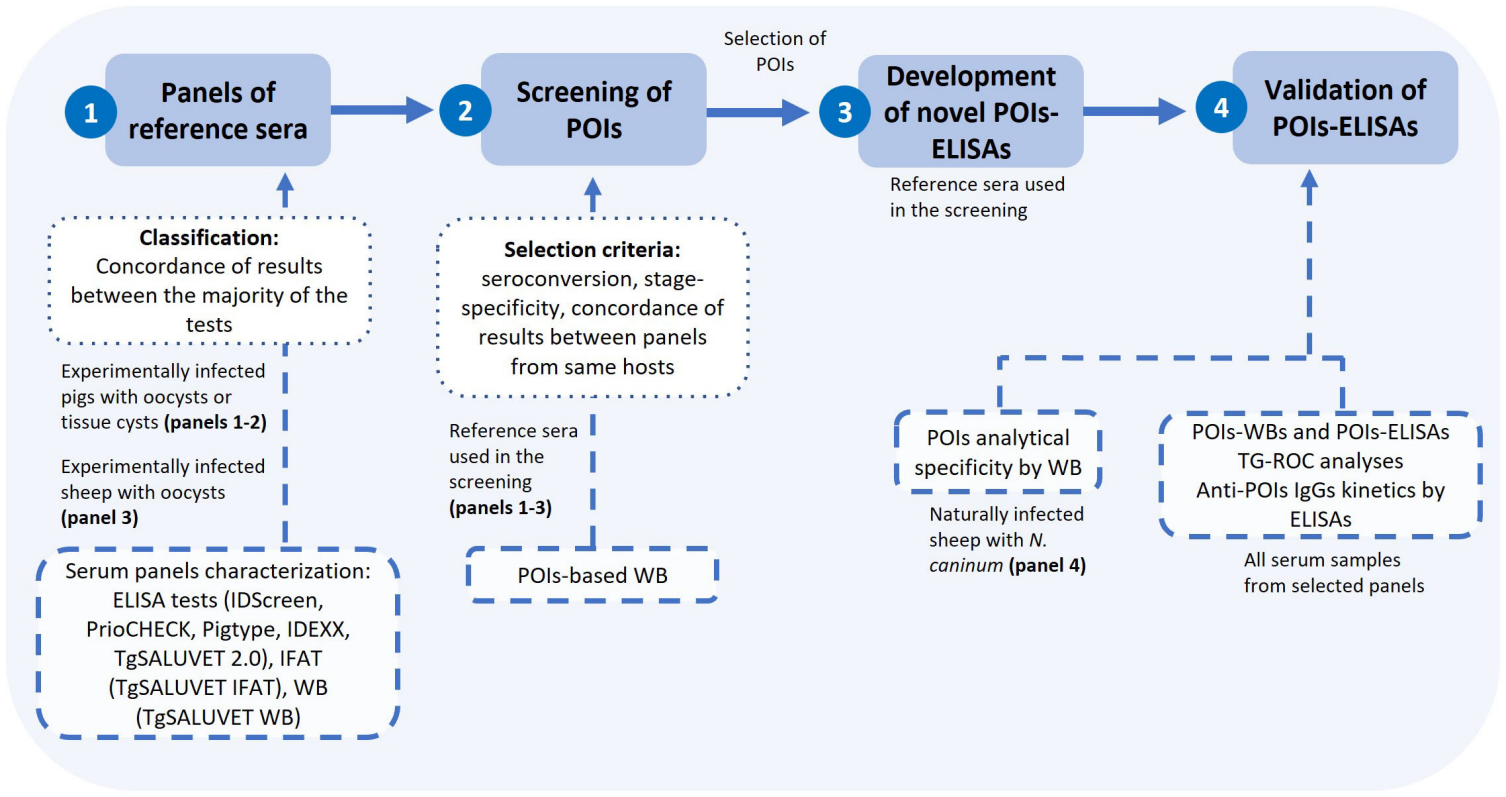
Workflow followed in the screening of proteins-of-interest from *T. gondii* oocysts and the enzyme-linked immunosorbent assay (ELISA) development. IDScreen: ID Screen® Toxoplasmosis Indirect Multi species; PrioCHECK: PrioCHECK® Porcine Toxoplasma Ab Kit or PrioCHECK® Toxoplasma Ab SR; Pigtype: Pigtype® Toxoplasma Ab from Indical Bioscience/Qiagen; IDEXX: IDEXX Toxotest Ab; IFAT: immunofluorescence antibody test; WB: Western blot test; POIs: proteins-of-interest.

We followed a recommended workflow for the screening of *T. gondii* oocyst stage-specific antigens aiming at the development of a serological tool with source-attribution usefulness (Álvarez García et al., 2021). The experimental design is detailed in **Figure 2**. Three panels of sera from either experimentally infected pigs (panels 1 and 2) or sheep (panel 3), previously well-characterized by a battery of conventional serological tests (Largo-de la Torre et al., 2022; López-Ureña et al., 2023a; López-Ureña et al., 2023b; **Supplementary Figure 2**, and **Supplementary Table 3**), were used. First, a selection of pig and sheep sera were used as reference for the screening of the POIs by WB: three pigs infected with oocysts and two pìgs infected with tissue cysts, up to 3 weeks post-infection (wpi) (panel 1), ii) one non-infected pig and two pigs infected with oocysts, up to 42 days post-infection (dpi) (panel 2) and iii) one non-infected sheep and two sheep infected with oocysts, up to 21 dpi (panel 3). For both pig and sheep reference sera, POIs were considered as putative candidates for source-attribution based on two main criteria: i) the detection of seroconversion (reactivity exclusively after the infection) and ii) reactivity to a protein band compatible with the molecular weight predicted for the recombinant polypeptide. Further criteria for antigen selection using pig sera were the ability to differentiate between oocyst- *vs.* tissue cysts-driven infection and the concordance between the two pig serum panels. Selected POIs were used to develop and optimize an ELISA test. Then, the whole set of samples from serum panels were analyzed by POI-based WBs and the newly developed POI-based ELISAs, and POI-based WBs were regarded as reference for a TG-ROC analyses and further POI-based ELISAs standardization. After analyzing the pattern of recognition by both POI-based techniques using all serum samples from selected panels, a POI was regarded as environmental source-attributing if it continued to be recognized only by animals infected with oocysts after the infection (up to 6 wpi for pig sera from panel 1, 42 dpi for pig sera from panel 2 and 21 dpi for sheep sera from panel 3). The kinetics of anti-POIs IgGs was later studied. In parallel, cross-reactivity of anti-*Neospora caninum* antibodies (IgGs) was tested by the POI-based WBs (**Figure 2**).

#### 2.4.1 Serum panels

The experimental infections were performed for unrelated previous studies under the corresponding regulations (panel 1: approved by the Ministry of Education, Youth and Sports from Czech Republic, PP 55/2016; panel 2: approved by the Animal Welfare Committee of the Community of Madrid from Spain, PROEX 293.7/20, PROEX 290.4/20 and PROEX 062/19; panel 3: approved by the Animal Ethic Committee from the Spanish National Research Council (CSIC), 1063/2021). In the absence of a gold standard for the detection of anti-*T. gondii* IgGs in pigs and sheep, multiple commercial and in-house conventional serological tests were used for the characterization of the different available panels of sera (Largo-de la Torre et al., 2022; López-Ureña et al., 2023a; López-Ureña et al., 2023b; **Supplementary Figure 2**, and **Supplementary Table 3**). Commercial and in-house serological tests were performed as specified by the manufacturers and López-Ureña et al. (2023a; 2023b), respectively. Different host- and parasite-dependent factors were considered as they can greatly influence the immune response elicited by parasite antigens: two parasite infective stages (sporulated oocysts and tissue cysts), different inoculation doses, parasite strains, animal breeds and ages. Samples were classified as positive or negative based on the majority criteria (result obtained by most of the tests), seroconversion was recorded at 2-3 wpi and higher antibody levels corresponded to groups inoculated with type III isolates (Largo-de la Torre et al., 2022; López-Ureña et al., 2023a; López-Ureña et al., 2023b; Vallejo et al., 2023).

Panel 1 consisted of orally inoculated prepubertal sows with 400 *T. gondii* oocysts (n= 6) or 10 tissue cysts (n= 7) from type II isolate (CZ-Tiger strain, ToxoDB RFLP genotype #3), and 400 *T. gondii* oocysts (n= 6) or 10 tissue cysts (n= 6) from type III isolate (Šimková strain, ToxoDB#2, isolated from feces of a domestic cat (*Felis catus*), Dámek (2023)), respectively. Blood samples were collected weekly (from 0 to 6 weeks), with a few exceptions when the samples were not collected in all sampling weeks (n= 158). This panel was previously characterized by López-Ureña et al. (2023a), and based on the results from most of the tests from that study, seroconversion was recorded from 2 wpi (positive: n= 97, negative: n= 48, doubtful; n= 13). In addition, all serum samples were analyzed by a WB based on soluble antigen from sporulated oocysts, and seroconversion was also recorded from 2 wpi in all experimental groups (unpublished data). This panel was used for the screening of POIs and the development of the novel POI-based ELISAs. For the screening of POIs and the development of the POI-based ELISAs, sera from three and two pigs infected with oocysts (type II and III) or tissue cysts (type III), respectively, from 0, 1, 2 and 3 wpi, were selected.

Panel 2 was composed of non-infected (n= 3) and orally inoculated three-months-old female pigs with 1,000 oocysts from the isolates TgShSp1 (type II, ToxoDB#3, n= 5) and TgShSp24 (type III, ToxoDB#2, n= 5), bled at -4, 0, 4, 7, 14, 21, 28 and 42 dpi (n= 103) (Largo-de la Torre et al., 2022). This panel of sera was characterized within this study (**Supplementary Figure 2**) and pigs seroconverted from 14 dpi by most of the serological tests (positive: n= 40, negative: n= 63, and no doubtful results). This panel was used for the screening of POIs, the development of POI-based ELISAs and further analyses. For the screening of POIs and the development of the POI-based ELISAs, sera from one non-infected and two infected pigs (with both isolates), from all sampling days, were used.

Panel 3 consisted of sera from non-infected (n= 6) and experimentally infected pregnant sheep (n= 14) orally inoculated with 10 *T. gondii* oocysts from the isolate TgShSp1 (type II, ToxoDB #3, n= 124) bled at -2, 3, 8, 12, and 14 dpi, with 6 of these sheep also sampled at 21 and 27 dpi (Vallejo et al., 2023). This panel of sera was characterized by López-Ureña et al. (2023b) and seroconversion was recorded from 21 dpi based on most of the tests (positive: n= 18, negative: n= 106, and no doubtful results). It was used for the screening of POIs, the development of POI-based ELISAs and further analysis. Sera from one non-infected and two infected sheep from -2, 8, 14 and 21 dpi were used for the screening of the POIs and the development of POI-based ELISAs.

Panel 4 consisted of twenty-three serum samples from sheep naturally infected with *N. caninum* from semi-intensive flocks. All these samples tested positive by a *N. caninum* tachyzoite-based WB under reducing conditions and a *N. caninum* tachyzoite soluble antigen-based ELISA, but were found negative by a *T. gondii* lyophilized tachyzoite-based ELISA and a *T. gondii* tachyzoite based-WB under reducing conditions (Sánchez-Sánchez et al., 2021). This panel of sera was used to test for cross-reactivity of selected POIs with anti-*N. caninum* IgGs by POIs based-WBs. The sera employed were grouped based on the level of anti-*N. caninum* IgGs as follows: low (RIPC= 37-60, n= 5), medium (RIPC= 61-89, n= 10), and high (RIPC≥ 90, n= 8).

#### 2.4.2 Protein-of-interest-based Western blot tests

All POIs were quantified using the Bradford method (Bio-Rad), following manufacturer’s instructions. Appropriate amounts (5-15 µg) of each POI were resuspended in loading buffer (10% of glycerol, 50 mM of TRIS pH 6.8, 2% of SDS, 0.05% of bromophenol blue and 100 mM of DDT), and incubated at 100°C for 5 min. Following resolution by SDS-PAGE on one-comb 15% polyacrylamide gels along with a pre-stained protein standard (Precision Plus Protein™ Kaleidoscope™, Bio-Rad), the POIs were transferred to a 0.2 µm nitrocellulose membrane and visualized by staining with 0.1% Ponceau Red (Sigma). Then, the membranes were blocked for 2 h at room temperature (RT) with 5% of skimmed milk in 0.05% TBS-Tween 20 (TBS-T), washed three times, each for 5 min, and kept at -20°C until use. For the screening of serum panels, samples were diluted 1/20 in blocking solution and incubated for 1.5 h at RT (1-2 mm membrane strips were used). After three washes with TBS-T, each for 5 min, peroxidase-conjugated secondary reagents were added (pigs: peroxidase-conjugated protein G at 1/600 (Sigma); sheep: peroxidase-conjugated monoclonal anti-goat/sheep IgG at 1/1,000 (Sigma)) and incubated for 1.5 h. Then, the strips were washed as described above, and finally with TBS. Bound primary antibodies were visualized colorimetrically using a 4-chloro-1-naphthol solution (Thermo Scientific). The reaction was stopped after 30 min of incubation at RT by adding ultra-pure water. The nitrocellulose strips were scanned using a GS-800 Calibrated Densitometer (Bio-Rad) for further analysis. In the case of TgOWP8, one important limitation was the recognition of multiple bands, which hampered the interpretation of the results. Thus, we only considered the recognition of a 70 kDa band corresponding to TgOWP8 predicted molecular weight.

#### 2.4.3 Protein-of-interest-based ELISAs

After testing several experimental variables, the ELISA conditions yielding the better discrimination between reference serum samples before and after infection were selected. Briefly, 100 µL/well of up to 4 µg/mL of individual POIs (TgCCp5A and TgSR1) diluted in cold PBS were used to coat 96 wells plates (MaxiSorp™, Thermo Scientific) overnight at 4°C. Then, the plates were washed three times with 0.05% PBS-Tween 20 (PBS-T) and blocked with 300 µL/well of 5% w/v powdered skim milk in PBS-T for 2 h at RT. The plates were subsequently washed and 100 µL/well of 1/100 diluted serum samples in blocking solutions were incubated for 1 h at 37 °C. Following three washes, 100 µL/well of 1/3,000 diluted peroxidase-conjugated Protein G in PBS-T were added. The plates were incubated and washed as described above and then 100 µL/well of TMB Ultra were added (Thermo Scientific). The reaction was stopped after 10 min of RT incubation by adding 100 µL of 2 N sulfuric acid, and optical densities were immediately read at 450 nm using a microplate reader (Multiscan RC 6.0, Labsystems). Positive and negative controls were selected based on the results from the screening of WBs from each selected POI. Results were interpreted as relative index percent (RIPC): ([sample OD-negative control OD]/[positive control OD-negative control OD]) x 100.

The average coefficient values (CV) of the intra- and inter-plate repeatability of the CCp5A-ELISA were below 5 (standard deviation (SD)= 0.01). TgCCp5A-ELISA showed an area under the curve (AUC) of 0.82 with 80% sensitivity (Se) and 74% specificity (Sp) for the selected cut-off, RIPC≥ 23.69, using as reference TgCCp5A-WB (TgCCp5A-WB results, positive: n= 55, negative: n= 206). For the TgSR1-ELISA, the mean CV values of intra- and inter-plate repeatability were also below 5 (SD= 0.001). TgSR1-ELISA showed an area under the curve (AUC) of 0.97 with 100% Se and 85% Sp for the cut-off selected, RIPC≥ 24.78, using as reference TgSR1-WB (TgSR1-WB results, positive: n= 17, negative: n= 243).

### 2.5 Data analysis

For the precision (intra- and inter-plate variability) of POI-based ELISAs, positive and negative controls were tested in triplicate, with three different plates for each selected POI. The coefficient of variation (CV) was determined as follows: mean ([standard deviation of the three replicate ODs/mean of the three replicate ODs] x 100), or mean ([standard deviation of the OD mean of each sample from each plate/mean of the OD mean of each sample from each plate] x 100). The POI-ELISAs cut-off values were defined with a non-parametric two-graph receiver operating characteristic (TG-ROC) analysis, performed with SigmaPlot 12.0, using as reference test the POI WB.

Anti-POIs IgG kinetics were studied with serum panels from experimental infections based on the defined cut-off values, as well as based on statistically significant differences. For the statistical analysis, all animals that tested positive for the POIs prior to infection, based on the established cut-off values for each ELISA, were discarded. A two-way ANOVA or a mixed-effects analysis with multiple comparisons and repeated measures was performed with two different approaches: i) comparing the mean RIPC of each sampling day/week after the infection with respect to the RIPC prior to infection within each experimental group to confirm seroconversion; ii) comparing the mean RIPC between experimental groups within each sampling day to confirm if there were differences based on the stage and isolate of *T. gondii.* When applied, the analysis was followed by a Tukey test. These analyses were done using GraphPad Prism, version 8.0.1, and differences were considered statistically significant when *P* values were lower than 0.05. The same approach was followed for the characterization of sera from panel 2, which included the kinetics of anti-*T. gondii* IgGs by different conventional ELISA tests (**Supplementary Figure 2**).

## 3 Results

### 3.1 *In silico* selection of antigen candidates

We filtered our previously curated set of 8,284 annotated proteins (Álvarez García et al., 2021) taken from ToxoDB (www.toxodb.org) (Harb and Roos, 2020) for oocyst-specific proteins which were absent in available tachyzoite and bradyzoite proteome datasets and which should be either secreted or surface-exposed and of relatively high abundance (**Figure 1**). This resulted in 331 proteins, of which 90 had a predicted signal peptide (determined with SignalP 5.0). After manual curation, five more sequences were added, resulting in 95 candidate proteins (**Supplementary Table 1**). They contained three antigens that have previously been reported to distinguish oocyst- from tissue cyst-derived infection, e.g. TgERP (TGME49_276850) (Hill et al., 2011); TgCCp5A (TGME49_258400) (Santana et al., 2015) and TgOWP8 (TGME49_271590) (Liu et al., 2019). In a final step we prioritized their cloning and expression according to their putative abundance in oocysts, using respective data from proteomic and transcriptomic studies when available (Fritz et al., 2012a; Fritz et al., 2012b). Finally, we evaluated the presence of predicted linear B cell epitopes using two algorithms, BepiPred2 (Jespersen et al., 2017) and EpiDope (Collatz et al., 2021). However, this was not followed further (see Discussion section) and did not influence our prioritization.

Using as templates *T. gondii* genomic DNA or a cDNA library from partially sporulated oocysts, we attempted PCR amplification of the full-length coding sequences, or parts thereof, of 83 out of the 95 selected genes. Of these 83 candidates, 50 (60%) did not reach the serological screening phase, either because of PCR failure or due to no/very low bacterial expression level. The purification of bacterially expressed proteins in sufficient amounts for serological analysis was successfully achieved for 32 antigens (see section 3.2. and **Table 1**).

**Table 1:** Serological screening of proteins-of-interest from *T. gondii* oocysts using serum samples from pigs and sheep experimentally infected with *T. gondii* oocysts and tissue cysts.

Gene ID	ToxoDB annotation	Localization	Screening by antigen-based WB in pigs and sheep The number of reactive animals is indicated in parenthesis for each group (*see legend for color interpretation†*)								# of amino acids of full length protein	position of the 6-histidine tag	Reference
			Pigs (Panel 1)				Pigs (Panel 2)		Sheep (Panel 3)				
			Oocysts (n= 3)		Tissue cysts (n= 2)		Oocysts (n= 2)		Oocysts (n= 2)				
			No recognition before Inf.?	Recognition after Inf.?	No recognition before Inf.?	Recognition after Inf.?	No recognition before Inf.?	Recognition after Inf.?	No recognition before Inf.?	Recognition after Inf.?			
TGME49_204420	TgOWP1	Oocyst wall	†	(1)		†					499 (25-252)	N	Possenti et al., 2010
TGME49_209610	TgOWP2	Oocyst wall					(1)	(2)		(1)**	462 (26-291)	N	Possenti et al., 2010
TGME49_271590	TgOWP8	Oocyst wall		(1)	(2)						229 (24-229)	N	Salman et al., 2017
TGME49_223700	F5/8 type C domain-containing protein	Sporozoite cytoplasm	(2)	(2)		(2)	(1)	(1)		(2)**	1571 (21-335)	N	Spano F., unpublished
TGME49_256040	PA14 domain-containing protein	Sporozoite cytoplasm			(1)	(1)					998 (501-998)	N	Spano F., unpublished
TGME49_258400	TgCCp5A	Sporozoite cytoplasm		(2)				(1)	(1)	(1)	996 (555-996)	N	Spano F., unpublished
TGME49_258550	TgSAG-related protein TgSRS28	Sporozoite surface	(1)	(1)		(2)		(2)	(1)	(1)	291 (25-291)	N	Radke et al., 2004
TGME49_259670	von Willebrand factor type A domain-containing protein	NA									1080 (45-424)	N	NA
TGME49_267410	Scavenger receptor protein TgSR1 precursor (TgSR1 C-term)	Sporozoite cytoplasm		(2)				(1)		(1)**	1245 (761-1245)	N	Spano F., unpublished
TGME49_268310	TgOWP3	Oocyst wall				(1)	(1)	(1)		(1)**	640 (71-477)	N	Possenti et al., 2010
TGME49_276850	Late embryogenesis abundant protein (TgERP)	NA						(1)			104 (1-104)	C	Hill et al., 2011
TGME49_276860	Late embryogenesis abundant protein	NA	(2)	(2)	(1)	(2)			(2)	(2)	517 (27-517)	C	Fritz et al., 2012a
TGME49_276870	Late embryogenesis abundant protein	NA									171 (1-171)	C	Fritz et al., 2012a
TGME49_276880	Late embryogenesis abundant protein	NA									130 (1-130)	C	Fritz et al., 2012a
TGME49_315730	Apical membrane antigen 3 (TgAMA3)	Sporozoite micronemes				(2)					651 (393-566)	N	Fritz et al., 2012b
TGME49_205090	Toxoplasma family D protein (TgOWP11)	Oocyst wall*									575 (20-575)	N	Salman et al., 2017
TGME49_222940	Hypothetical protein	Oocyst wall*	(1)	(2)	(1)	(1)	(1)	(1)			489 (48-489)	N	NA
TGME49_236975	*Toxoplasma* family D protein	Oocyst wall*	(2)	(3)	(1)	(1)		(2)	(1)	(1)	582 (21-582)	N	NA
TGME49_272240	*Toxoplasma* family D protein (OWP12)	Oocyst wall*									634 (28-634)	N	Salman et al., 2017
TGME49_316560	*Toxoplasma* family D protein	Oocyst wall*									569 (22-569)	N	NA
TGME49_316670	*Toxoplasma* family D protein	Oocyst wall*	(1)	(1)	(1)	(1)					644 (26-644)	N	NA
TGME49_209920	PAN-domain containing protein	NA							(1)	(2)	991 (17-991)	N	NA
TGME49_295640	Peptidase family M13 protein	NA					(1)	(2)			1038 (32-1038)	N	NA
TGME49_262470	C protein immunoglobulin-A-binding beta antigen	NA							(2)	(2)	303 (24-303)	C	NA
TGME49_204520	Hypothetical protein	NA									369 (20-369)	N	NA
TGME49_270950	Hypothetical protein	NA						(1)			357 (22-357)	C	NA
TGME49_292350	Hypothetical protein	NA			(1)	(2)		(1)	(2)	(2)	169 (21-169)	C	NA
TGME49_287250	Hypothetical protein	NA									222 (25-222)	N	NA
TGME49_254780	Hypothetical protein	NA									112 (23-112)	N	NA
TGME49_244260	Hypothetical protein	NA									167 (18-167)	N	NA
TGME49_273705	Hypothetical protein	NA									424 (27-424)	N	NA
TGME49_316890	Hypothetical protein	NA									407 (24-407)	N	NA

†Cells in green indicate afirmative answer to the column question and white cells indicate a negative answer, thus two consecutive cells in oocysts-infected groups is an expected result for an oocyst-specific marker as long as there is a white cell in the "recognition after the infection" column for the tissue cytst-infected group; *Putative localization; ** Serum samples from the non-infected sheep (negative control) also showed reactivity to these antigens, reason why they were not selected; NA: not available; WB: Western blot test.

### 3.2 Selection of two putative oocyst-specific serological markers by applying our criteria

A total of 32 POIs were initially screened by WB with the subset of experimentally infected animals. Most of the POIs were discarded for the following reasons: i) they were not recognized at any sampling day by WB, ii) they were recognized prior to infection, iii) they did not discriminate between oocysts- and tissue cysts-driven infections, and/or iv) they showed discrepant reactivity with pig sera from panels 1 and 2 (see section 2.4.1 for panel description) (**Table 1**). Noteworthy, applying these criteria we excluded four proteins from subsequent experimental steps, which had been previously reported as antigens with source-attributing potential, i.e., TgERP (LEA850) (Hill et al., 2011; Vieira et al., 2015; Burrells et al., 2016; Mangiavacchi et al., 2016), TgSporoSAG (Crawford et al., 2010; Döşkaya et al., 2014), TgOWP1 (Santana et al., 2015) and TgOWP8 (Liu et al., 2019). The recombinant proteins TgCCp5A and TgSR1 reacted stage-specifically with pig sera from both panels (2/3 pigs from panel 1 and 1/2 pigs from panel 2) only after infection, indicating seroconversion of the animals (**Table 1**). In contrast, none of the POIs passed the initial screening when using sera from sheep (panel 3) (**Table 1**). Based on these results, TgCCp5A and TgSR1 were selected for the development of a source-attributing ELISA for pigs.

### 3.3 TgCCp5A and TgSR1 lack source-attribution properties

**Figure 3:**
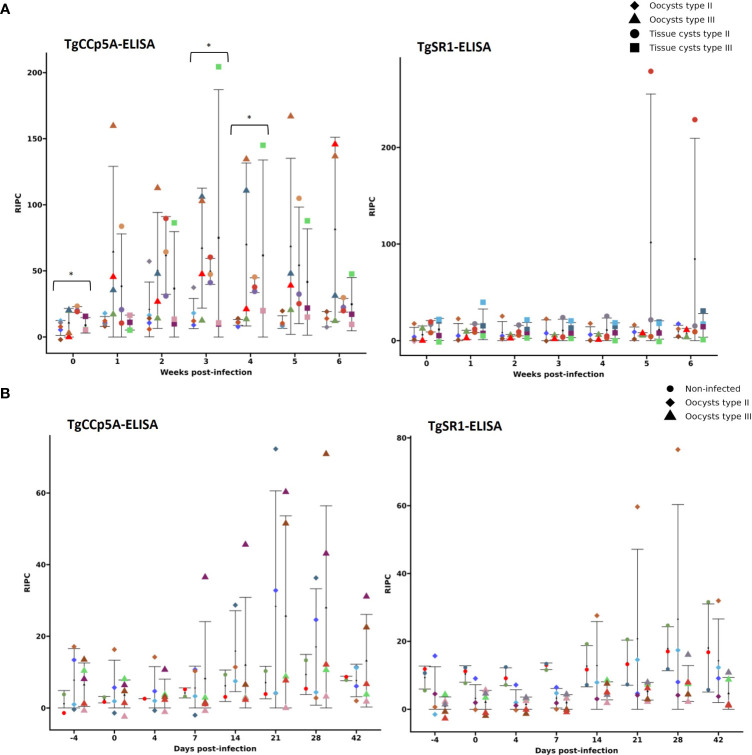
Kinetics of anti-TgCCp5A and anti-TgSR1 IgGs in serum panels 1 **(A)** and 2 **(B)** based on the novel developed enzyme-linked immunosorbent assays (ELISAs). Data presented as the mean of the relative index percentage (RIPC) by experimental group ± the standard deviation (SD). Each color represents one pig through the experiment within each graph. A pig color could vary between graphs, but not within graphs. Significant differences between groups infected with oocysts vs. tissue cysts within sampling weeks/days from each ELISA are identified as follow: *= *P*< 0.05. When these panels were analyzed by TgCCp5A-WB, 36.5% (4/11) and 58.3% (7/12) of animals infected with oocysts and tissue cysts from panel 1 showed positive reactivity, respectively, *vs.* 28.6% (2/7) of pigs infected with oocysts from panel 2 (pigs with TgCCp5A-WB-positive reactivity prior to infection were excluded). Based on TgSR1-WB, 18.2% (2/11) and 25% (3/12) of pigs infected with oocysts and tissue cysts from panel 1 tested positive, respectively, *vs.* 10% (1/10) of infected pigs from panel 2 (TgSR1-WB-positive animals prior to infection were also excluded).

To quantify serological responses, we established a specific TgCCp5A-ELISA (see section 2.4.3). The analysis of all serum samples from both pig panels (n= 261) by TgCCp5A-ELISA and TgCCp5A-WB revealed a lack of stage specificity and low antigenicity of this POI, reflected by 28% (7/25) of pigs from panel 1 and 10% (1/10) from panel 2 giving positive results prior to infection. Thus, these samples were excluded from the seroconversion analysis. Seroconversion was recorded in 50% (5/10) and 67% (4/6) of pigs infected with oocysts and tissue cysts from panel 1, respectively, and in 44% (4/9) of infected pigs from panel 2. In addition, a high variability within experimental groups was observed: i) a few animals seroconverted to TgCCp5A; ii) the RIPC values remarkably varied within the groups (**Figure 3**). A similar scenario was observed with TgCCp5A-WB (see **Figure 3** description).

Notably, compared to prior infection, none of the experimental groups from panels 1 and 2 showed a significant increase of anti-TgCCp5A IgGs by ELISA throughout the sampling period, except for panel 1 at 4 wpi in pigs infected with type II tissue cysts. Considering differences between experimental groups within a sampling week, there was a significant higher level of anti-TgCCp5A IgGs in pigs infected with type II tissue cysts with respect to pigs infected with oocysts from the same isolate at 0, 3 and 4 wpi (*P*< 0.05) (**Figure 3**).

We also developed a TgSR1-ELISA (see section 2.4.3). When its cut-off was applied, 36% (9/25) of infected pigs from panel 1 and 10% (1/10) of infected pigs from panel 2 tested positive prior to infection. When these animals were excluded, 14% (1/7) of pigs from panel 1 infected with oocysts and 57% (4/7) of those infected with tissue cysts seroconverted, in contrast to 11% (1/9) of infected pigs from panel 2. A high variability among experimental groups was also observed, and a similar scenario was recorded with TgSR1-WB (see **Figure 3** description).

A non-significant increase of anti-TgSR1 IgGs was observed by SR1-ELISA in the different experimental groups after the infection (**Figure 3**).

### 3.4 Failure of previously described proteins with source-attribution properties to meet our criteria

In our study, TgERP consistently showed low immunogenicity and lacked stage-specific recognition. Only three pigs from panel 1 tested positive by TgERP-WB at 3 wpi, all of them had been infected with tissue cysts. Although two pigs from panel 2 reacted with TgERP at 4 dpi and 7 dpi, another five oocyst-infected pigs from the same panel reacted with TgERP prior to infection, as well as two non-infected pigs included in the negative control group.

Despite its higher antigenicity, TgSporoSAG was not considered suitable as a source-attributing indicator. In panel 1, 77.8% (7/9) and 72.7% (8/11) of pigs infected with oocysts or tissue cysts tested positive, respectively, from 1 wpi to 6 wpi. In panel 2, 100% (10/10) of infected pigs seroconverted from 4 dpi to 42 dpi. However, also one non-infected pig serum reacted with TgSporoSAG, starting at 21 dpi until the end of the experiment.

On the other hand, only 18.2% (2/11) and 16.7% (2/12) of pigs infected with oocysts or tissue cysts from panel 1 developed antibodies against TgOWP1 by WB, respectively, from 2 to 6 wpi, and 10% (1/10) of infected pigs from panel 2 seroconverted at 14 dpi.

TgOWP8 was recognized by 14% (1/7) of pigs from panel 1 infected with tissue cysts, while none of the pigs infected with oocysts tested positive. In addition, some pigs showed reactivity prior to infection. In panel 2, 37.5% (3/8) of infected pigs recognized TgOWP8, starting 4 dpi, and two infected and three non-infected pigs recognized TgOWP8 prior to infection.

## 4 Discussion

The difficulty to discriminate between infections from the meat-borne route *vs.* the environmental route is one of the most relevant gaps for the prevention and control of *T. gondii* infections from a One Health perspective. Stage-specific serology could be a game-changer to fill this knowledge gap. It could be applied to humans as well as animals along the food-chain, to inform interventions targeting the relevant transmission routes. Indeed, a few sporozoite- or oocyst/sporocyst wall-specific proteins have been suggested as putative diagnostic markers for this purpose (reviewed by Álvarez García et al., 2021). This study represents the first attempt, using strict guidelines for the validation of diagnostic tests (Newberry and Colling, 2021), to evaluate the source-attribution potential of previously described and novel predicted sporozoite- or oocyst/sporocyst wall-specific proteins.

At the beginning of this study we used the two most recent algorithms at the time for linear B-cell epitope prediction, BepiPred2 (Jespersen et al., 2017) and EpiDope (Collatz et al., 2021), for assessment of the antigenicity of our candidates. However, we found contradicting predictions by both methods for many proteins (data not shown). This prompted us to leave this approach aside for our selection scheme. However, the description of AlphaFold2 in late 2021 (Jumper et al., 2021) has changed the situation substantially, making the prediction of conformational epitopes feasible also for those putative antigens where only the primary amino acid sequence is known. Consequently, this resulted in the recent description of DiscoTope 3 (Høie et al., 2023), an algorithm that aims to identify conformational B-cell epitopes from AlphaFold2-predicted 3D protein structures. Therefore, we were interested to see retrospectively if our prioritization scheme would have been influenced by this and two other recently described methods. One is the ‘Antigenic Protein and Peptide Ranker’ (APRANK), aimed at prioritizing putative antigens of several pathogens, including *T. gondii*, based on *in silico* analyses (Ricci et al., 2021). The other is the recently improved BepiPred algorithm (V3) (Clifford et al., 2022). As explained in detail in **Supplementary Figures 1A–E**, our dataset of 95 candidates (**Supplementary Table 1**) showed that there is only little overlap (6 out of 95; **Supplementary Figure 1D**) where all three algorithms allowed the calculation of a epitope prediction score (cEPS), which allows the comparison of the algorithms (see Materials and Methods section). No significant correlations between the different scores could be observed (discussed in **Supplementary Figure 1**; and data not shown). In conclusion, we probably would have made the same (possibly false) decisions in case we had included Discotope 3, Bepipred 3 or EpiDope for our prioritization. Our results and those from other studies (Galanis et al., 2021; Batisti Biffignandi et al., 2023; Cia et al., 2023) indicate that despite recent improvements in the algorithms, making antigenicity predictions the basis for *a priori* selection of candidates for serological assays is of limited use. Their utility lies more in the verification of epitopes of known antigenic proteins.

There have been previous studies that reported on *T. gondii* antigen discovery using either protein (Liang et al., 2011; Felgner et al., 2015; Döşkaya et al., 2018) or peptide microarrays (Maksimov et al., 2012; Arranz-Solís et al., 2019; Arranz-Solís et al., 2021), but without a special emphasis on stage-specificity. None of the reactive polypeptides (exon products) of the protein arrays described by Döşkaya et al. (2018), which could still be retrieved from current ToxoDB (196 out of the previously reported 240 IDs) and that were probed with sera from mice either infected with tissue cysts or oocysts, met our selection criteria. Comparing the APRANK scores of these 196 proteins with those of our 95 candidates, also shows that their criteria (based on GO terms “outer membrane; heat-shock protein; chaperone; transport protein; integral membrane protein; transmembrane protein; lipoprotein or virulence associated protein” (Döşkaya et al., 2018)) resulted in inferior scores (**Supplementary Figure 1E**).

For specific diagnosis of oocyst-derived infections, herein a set of complementary *in silico* tools focused on the identification of predicted sporozoite- or oocyst/sporocyst wall-specific antigens. It resulted in a total of 32 candidates (POIs) that could be expressed and purified in sufficient amounts for further testing. We did not filter the candidates for being specific for *T. gondii* or other Coccidia (n= 48) (**Supplementary Table 1**), but rather chose to show their specificity during later steps of the experimental analysis once promising antigens would have been identified. Among those 32 proteins, five proteins tested in previous studies were included: TgERP, TgCCp5A, TgOWP1, TgSporoSAG and TgOWP8 (Crawford et al., 2010; Hill et al., 2011; Santana et al., 2015; Vieira et al., 2015; Burrells et al., 2016; Mangiavacchi et al., 2016; Liu et al., 2019).

We followed our very detailed and robust workflow to screen such antigens and develop an ELISA test able to identify oocyst-derived infections (Álvarez García et al., 2021). Accordingly, several limitations identified in earlier work were tackled systematically: i) the employment of different panels of reference serum samples from experimentally infected animals that were previously well-characterized through a battery of serological tests; ii) the use of a POI-based WB as a confirmatory test in combination with the development of a POI-based ELISA, and iii) the study of cross-reactions with the closely related Sarcocystidae parasite *N. caninum*. This restrictive criterion aimed at screening stage-specific immune responses in two relevant target species, pig and sheep. Sera from both are adequate reagents for testing the POI´s antigenicity since both species are relevant *T. gondii* IH. Moreover, pigs and humans share similar immunological features (Delgado Betancourt et al., 2019). This is the first study where sheep sera were used and where isolates of predominant archetypal types II and III were employed as inoculum in pigs. Moreover, up to now, studies that employed sera from experimental infections comparing both transmittable parasite stages are limited to one study carried out with pig sera (Hill et al., 2011) and three studies based on mouse sera (Crawford et al., 2010; Döşkaya et al., 2014; Santana et al., 2015). In addition, all serum panels used herein had been previously characterized, avoiding bias due to differences in diagnostic performance of tests (López-Ureña et al., 2023a; López-Ureña et al., 2023b).

In the initial screening, TgCCp5A and TgSR1 showed promising results with pig sera. However, only 1 or 2 out of up to 3 animals infected with oocysts from each panel detected the proteins (**Table 1**), indicating low immunogenicity. Accordingly, when TgCCp5A and TgSR1 were subjected to a more detailed analysis with the whole set of pig sera, the lack of stage-specificity and unspecific recognition by non-infected animals finally discredited these proteins as reliable source-attribution markers. In addition, all sera from *N. caninum*-infected sheep cross-reacted with TgCCp5A and TgSR1, which is not surprising given the marked conservation of both proteins across the Phylum Apicomplexa (**Supplementary Table 1**). Thus, testing cross-reactivity with closely related parasites is recommended in further studies. While TgSR1 was evaluated for the first time, reactivity of sera from different species (pig, chicken, and humans) against TgCCp5A was previously evaluated by others (Santana et al., 2015; Liu et al., 2019). Importantly, none of these studies had used sera from pigs experimentally infected with both parasite stages, which is a crucial control. In a previous study, sera from naturally infected pigs were reported to show a significant increase in anti-TgCCp5A IgGs at 7 and 14 dpi (Santana et al., 2015). Others found 12% (11/90) of positive field pigs reacting with TgCCp5A (Liu et al., 2019). However, the time post-infection and the parasite stage involved was unknown since serum samples were from animals with natural infections. Further shortcomings were a lack of reference tests for sera characterization (Santana et al., 2015; Liu et al., 2019) and a higher IgM response with soluble tachyzoite antigen compared to TgCCp5A. Although TgCCp5A was recognized by human IgM and IgG in a toxoplasmosis outbreak, this was not seen by all sera (Santana et al., 2015). In addition, Liu et al. (2019) provided data of reactive sera from human patients with unknown clinical and serological history.

On the other hand, TgERP, TgSporoSAG, TgOWP1 and TgOWP8, reported in the literature as antigens able to serologically identify oocyst-driven infections, were excluded from the initial screening. TgERP (also called TgLEA850; Arranz-Solís et al., 2023), has been employed in several studies in humans and raised hopes about its source-attributing potential (Hill et al., 2011; Vieira et al., 2015; Burrells et al., 2016; Mangiavacchi et al., 2016; EFSA Panel on Biological Hazards et al., 2018). However, in the present study, TgERP showed limited antibody recognition and lack of stage-specificity. Other authors previously showed that TgERP was not exclusively recognized in oocyst-derived infections in humans (Hill et al., 2011), providing limited evidence for environmental contamination with oocysts. In the same study, pig serology indicated recognition of TgERP by sera from animals infected with oocysts *vs.* tissue cysts (Hill et al., 2011). This contrasts with the low reactivity and lack of stage specificity observed in our study with this protein. A low immunogenicity might be explained by the animals breed and age, but also by the antigens’ time of exposure to the immune system as mentioned above. Moreover, a recent report has shown that TgERP is an intrinsically disordered protein, lacking a defined structure (Arranz-Solís et al., 2023), which might influence the immune response at the individual level (Uversky and Van Regenmortel, 2021).

In our hands, TgOWP1 and TgOWP8 also showed limited antibody recognition and lack of stage specificity. In contrast, two previous studies reported recognition of TgOWP1 by sera from free-range chickens as an indicator of oocyst-driven infections, although not for pigs infected with oocysts (Santana et al., 2015; Liu et al., 2019). In addition, TgSporoSAG was recognized by most infected pigs of panels 1 and 2, but it also failed to differentiate between oocyst- and tissue cyst-driven infections. A significant increase of anti-TgSporoSAG antibodies at 40 and 120 dpi in mice infected with oocysts was previously reported (Döşkaya et al., 2014), but the stage-specific response was not evaluated. This contrasts with a previous study which reported a lack of reactivity of TgSporoSAG when tested with sera from oocyst-infected mice or naturally infected humans (Crawford et al., 2010). The reasons for this discrepancy are unclear but could lie in the different expression systems employed to produce recombinant TgSporoSAG (bacteria *vs.* insect cells), possibly leading to differently folded protein forms.

Following the above-mentioned strict workflow, no antigen with source attributing value was identified. This could be due to the short time period before sporozoites cease to express stage-specific antigens and differentiate into tachyzoites and the non-replicative nature of the sporozoite itself, excluding “antigen amplification” and thus the boosting of the immune response (Fabian et al., 2021). A major strength of this work was the use of validated reference sera from experimentally infected animals, previously not available. Systematic step-by-step approaches, as the one followed here, are recommended for further studies before human serum samples can be used for final validation. Their drawback, as with any serum from natural infections, is that adults could have been exposed to different *T. gondii* stages during their lifetime, making analyses very complex. The ultimate goal would be the use of serological tests to detect human oocyst-driven infections (e.g. outbreaks, seroconversion in pregnant women) to prioritize intervention strategies.

The conclusion from this extensive work exploring source-attributing serology is that there is currently no antigen that allows robust estimates of the proportion of *T. gondii* infections acquired from oocysts by serological tests. This work provided solid new insights that can be used for further research and development of serological source-attributing approaches for *T. gondii*.

## Data availability statement

The original contributions presented in the study are included in the article/**Supplementary Material**. Further inquiries can be directed to the corresponding authors.

## Ethics statement

The animal study was approved by Serum Panel 1: Ministry of Education, Youth and Sports from Czech Republic, PP 55/2016; Serum Panel 2: Animal Welfare Committee of the Community of Madrid from Spain, PROEX 293.7/20, PROEX 290.4/20 and PROEX 062/19; Serum Panel 3: Animal Ethic Committee from the Spanish National Research Council (CSIC), 1063/2021. The study was conducted in accordance with the local legislation and institutional requirements.

## Author contributions

N-ML-U: Conceptualization, Data curation, Formal Analysis, Investigation, Methodology, Software, Validation, Visualization, Writing – original draft, Writing – review & editing. RC-B: Funding acquisition, Resources, Supervision, Writing – review & editing. BK: Resources, Writing – review & editing. SC: Investigation, Methodology, Writing – review & editing. AP: Investigation, Methodology, Writing – review & editing. FT: Investigation, Methodology, Writing – review & editing. SK: Investigation, Methodology, Writing – review & editing. CS: Investigation, Methodology, Writing – review & editing. SJ: Investigation, Methodology, Writing – review & editing. PJ: Funding acquisition, Project administration, Resources, Writing – review & editing. JR-C: Resources, Writing – review & editing. L-MO-M: Funding acquisition, Project administration, Resources, Supervision, Writing – review & editing. FSp: Conceptualization, Data curation, Formal Analysis, Funding acquisition, Investigation, Methodology, Project administration, Resources, Software, Supervision, Validation, Visualization, Writing – original draft, Writing – review & editing. FSe: Conceptualization, Data curation, Formal Analysis, Funding acquisition, Investigation, Methodology, Project administration, Resources, Software, Supervision, Validation, Visualization, Writing – original draft, Writing – review & editing. GÁ-G: Conceptualization, Formal Analysis, Funding acquisition, Methodology, Project administration, Resources, Supervision, Validation, Visualization, Writing – original draft, Writing – review & editing.

## Supplementary material

Supplementary Table 1 Bioinformatic data of 95 candidate proteins.

Supplementary Table 2 Primers used in this study.

Supplementary Table 3
*Toxoplasma gondii*-based serological techniques used for the characterization of the serum panels from pigs and sheep experimentally infected with *T. gondii* oocysts and tissue cysts.

Supplementary Figure 1 Epitope prediction of candidate proteins.

Supplementary Figure 2 Anti-*Toxoplasma* IgG kinetics in experimentally infected pigs with oocysts from type II and III isolates (panel 2) based on different enzyme-linked immunosorbent assays (IDScreen, PrioCHECK, Pigtype and TgSALUVET 2.0). Seroconversion was recorded from 14-21 days post-infection by all ELISA tests (significance not represented in the figures). Significant differences between experimental groups within sampling days are identified in the figures as follow for each ELISA: *= *P*< 0.05, **: *P*< 0.01, ***= *P*< 0.001 and *P*< 0.0001. Seroconversion was detected from 14-21 days post-infection and differences between pigs infected with oocysts from type II *versus* type III isolates were recorded only with TgSALUVET 2.0 at 42 days post-infection.
