# Supplementary material for: Limited value of current and new in silico predicted oocyst-specific proteins of Toxoplasma gondii for source-attributing serology
Source: Front Parasitol. 2023 Nov 27;2:1292322. doi: 10.3389/fpara.2023.1292322 (PMC11731929; doi:10.3389/fpara.2023.1292322)
Supplement: Supplementary Table 3 — Toxoplasma gondii-based serological techniques used for the characterization of the serum panels from pigs and sheep experimentally infected with T. gondii oocysts and tissue cysts. [file Table_3.docx]

**Supplementary Table 3.** *Toxoplasma gondii*-based serological techniques used for the characterization of the serum panels from pigs and sheep experimentally infected with *T. gondii* oocysts and tissue cysts.

| **Technique** | **Antigen** | **Target species** | **Sample type** | **Sample dilution: type** |
| --- | --- | --- | --- | --- |
| IDScreen | SAG1 (P30) | Ruminants, pigs, dogs, cats | Serum, plasma and meat juice | Serum and plasma: 1/10, meat juice: 1/2 |
| PrioCHECK- small ruminants | Tachyzoites* | Small ruminants | Serum, plasma and meat juice | Serum and plasma: 1/100, meat juice: 1/10 |
| PrioCHECK)- porcine | Tachyzoites* | Pigs | Serum, plasma and meat juice | Serum and plasma: 1/50, meat juice: 1/10 |
| Pigtype | Tachyzoites* | Cattle, sheep, goats, cats, dogs, foxes, pigs and wild boars | Serum, plasma (meat juice just for pigs and wild boars) | Serum and plasma: 1/100, meat juice: 1/10 |
| IDEXX | Not specified** | Small ruminants | Serum and plasma | 1/400 |
| TgSALUVET ELISA 2.0 | Lyophilized tachyzoites | Sheep, goats, pigs | Serum | 1/100 |
| TgSALUVET IFAT | Tachyzoites | Pigs | Serum | 1/200 |
| TgSALUVET WB*** | Tachyzoites | Sheep, goats, pigs | Serum | 1/20 |

IDScreen: ID Screen® Toxoplasmosis Indirect Multi species ELISA, PrioCHECK: PrioCHECK® Toxoplasma Ab SR or PrioCHECK® Porcine Toxoplasma Ab ELISA kit, Pigtype: Pigtype® Toxoplasma Ab ELISA from Indical Bioscience/ Qiagen, IDEXX: IDEXX Toxotest Ab ELISA, IFAT: immunofluorescence antibody test, WB: Western Blot. *No more details provided. ** Just specified the microtiter plates are coated with inactivated antigen. ***Under reducing conditions.
