# Supplementary material for: Limited value of current and new in silico predicted oocyst-specific proteins of Toxoplasma gondii for source-attributing serology
Source: Front Parasitol. 2023 Nov 27;2:1292322. doi: 10.3389/fpara.2023.1292322 (PMC11731929; doi:10.3389/fpara.2023.1292322)
Supplement: Supplementary Figure 1 — Epitope prediction of candidate proteins. [file Image_1.pdf]

Supplementary Figure 1A-E: Epitope prediction of candidate proteins

Supplementary Discussion

Using the 16 *T. gondii* training antigens used by Ricci et al., complemented by 3 more known antigens used successfully in serological assays (TgBAG1, TgMAG1, TgGRA8 (Di Cristina et al., 2004; Pfrepper et al., 2005)), we first analyzed these 19 antigens with the three algorithms (DiscoTope 3, Bepipred 3, EpiDope) and the APRANK scores of the *T. gondii* proteome. We also included consensus protein disorder, based on the predictions from MobiDB (https://mobidb.bio.unipd.it/) (Piovesan et al., 2020), as described previously (Arranz-Solís et al., 2023). Note that all 19 antigens are not oocyst-specific and were thus not considered candidates. Since the outcomes of all three algorithms are a numeric value for each aa plotted along the protein sequence (see **S1 A**), we calculated a single value that should reflect the overall presence and abundance of putative antigenic sites and thus overall antigenicity, allowing the comparison of many proteins within the different algorithms (see Materials and Methods, and **S1 B** for details). It is similar to the APRANK score but, with the exception of BepiPred V1, is based on different predictors (Ricci et al., 2021) and calculated separately for each algorithm. Higher cEPS values are better.

Comparing our calculated cEPS for Discotope3, BepiPred 3, Epidope and Disorder of these 19 proven antigens to each other (data not shown) or with APRANK scores **(S1 C)**, we could not find any correlations, indicating that even proven antigens like GRA8, MIC8, MAG1 and ROP1, depending on the chosen predictor, would not have been prioritized over the others. Based on these results and not surprisingly, applying the same analyses to our dataset of 95 candidates **(Table S1)** showed that there is only little overlap where all three algorithms allowed the calculation of an cEPS (6 out of 95; **S1 D**) and no correlations between the different scores was observed (data not shown). Of course, lowering the thresholds or decreasing the number of consecutive aa (we used a quite short epitope length of 6 aa) would result in more common hits but at the expense of selectivity. However, the majority of our 95 candidates has a significantly higher APRANK score than the majority of the sequences of *T. gondii*'s proteome **(S1 E)**.

Arranz-Solís, D., Warschkau, D., Fabian, B.T., Seeber, F., and Saeij, J.P.J. (2023). Late Embryogenesis Abundant Proteins Contribute to the Resistance of Toxoplasma gondii Oocysts against Environmental Stresses. *mBio* 14(2)**,** e0286822. doi: 10.1128/mbio.02868-22.

Di Cristina, M., Del Porto, P., Buffolano, W., Beghetto, E., Spadoni, A., Guglietta, S., et al. (2004). The Toxoplasma gondii bradyzoite antigens BAG1 and MAG1 induce early humoral and cell-mediated immune responses upon human infection. *Microbes Infect* 6(2)**,** 164-171. doi: 10.1016/j.micinf.2003.11.009.

Pfrepper, K.-I., Enders, G., Gohl, M., Krczal, D., Hlobil, H., Wassenberg, D., et al. (2005). Seroreactivity to and avidity for recombinant antigens in toxoplasmosis. *Clin Diagn Lab Immunol* 12(8)**,** 977-982. doi: 10.1128/CDLI.12.8.977-982.2005.

Piovesan, D., Necci, M., Escobedo, N., Monzon, A.M., Hatos, A., Mičetić, I., et al. (2020). MobiDB: intrinsically disordered proteins in 2021. *Nucleic Acids Res* 54**,** 26. doi: 10.1093/nar/gkaa1058.

Ricci, A.D., Brunner, M., Ramoa, D., Carmona, S.J., Nielsen, M., and Agüero, F. (2021). APRANK: Computational Prioritization of Antigenic Proteins and Peptides From Complete Pathogen Proteomes. *Front Immunol* 12**,** 702552. doi: 10.3389/fimmu.2021.702552.

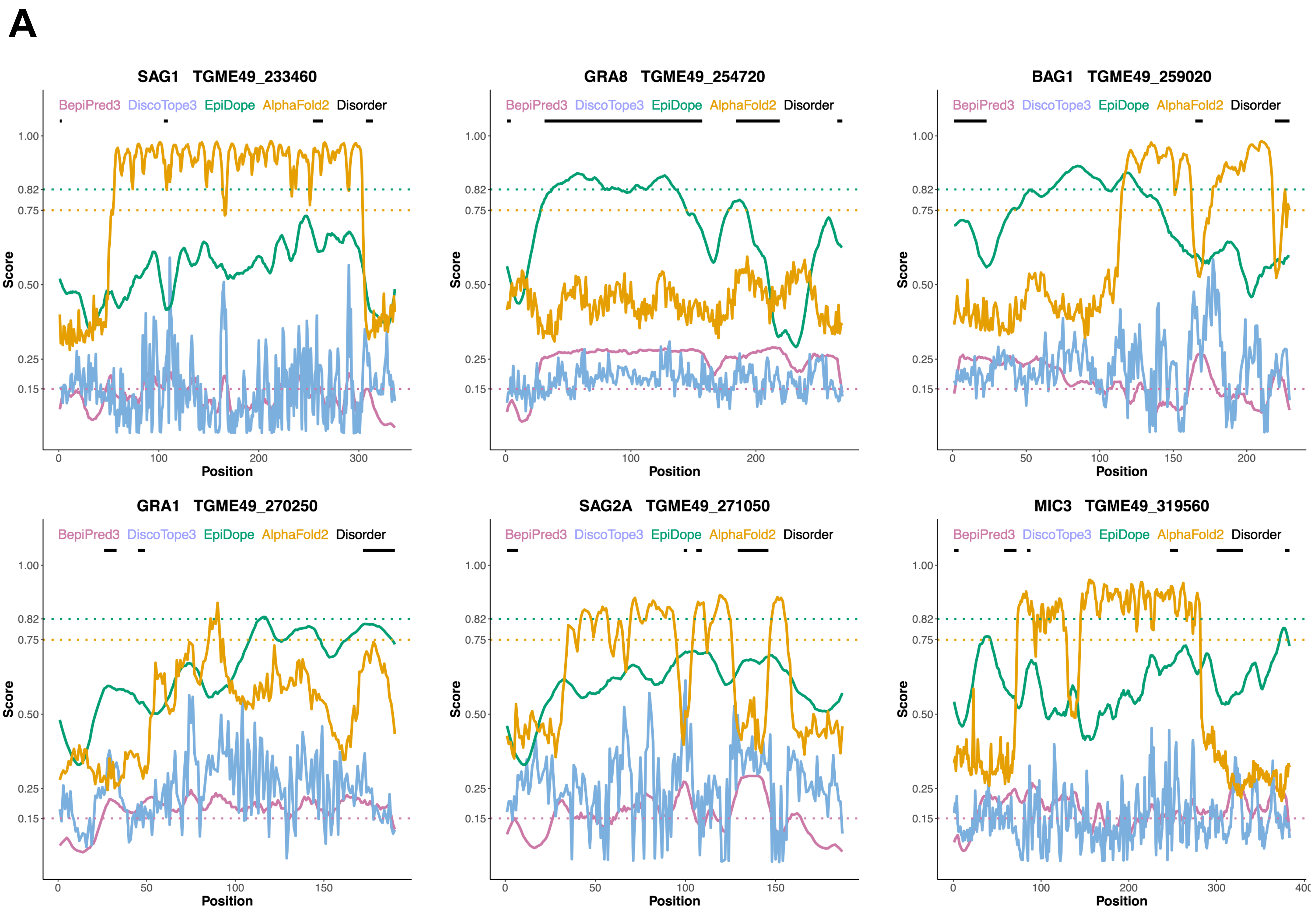
A
SAG1 TGME49_233460
GRA8 TGME49_254720
BAG1 TGME49_259020
GRA1 TGME49_270250
SAG2A TGME49_271050
MIC3 TGME49_319560
BepiPred3 DiscoTope3 EpiDope AlphaFold2 Disorder
Score
Position
1.00
0.82
0.75
0.50
0.25
0.15

B

Gene ID	Name	protein length	APRANK score[1]	EpiDope (t=0.82 [2])					BepiPred 3 (t=0.152)					DiscoTope 3 (t=0.25)					Disorder (t=1.05)			
				c[3]	d[4]	c/d[5]	%[6]	cEPS[7]	c	d	c/d	%	cEPS	c	d	c/d	%	cEPS	c	d	%	cEPS
TGME49_203310	GRA7	236	0.84	4.95	6	0.83	2.54	2.10	39.03	152	0.26	64.41	16.54	2.41	7	0.34	2.97	1.02	19.80	132	55.93	8.39
TGME49_227280	GRA3	220	0.45	20.94	25	0.84	11.36	9.52	27.87	124	0.22	56.36	12.67	20.46	66	0.31	30.00	9.30	10.20	68	30.91	4.64
TGME49_227620	GRA2	185	0.69						24.84	95	0.26	51.35	13.43	21.63	66	0.33	35.68	11.69	15.00	100	54.05	8.11
TGME49_233450	SRS29A	420	0.80	22.60	27	0.84	6.43	5.38	21.76	87	0.25	20.71	5.18	1.15	4	0.29	0.95	0.27	16.50	110	26.19	3.93
**TGME49_233460**	**SAG1**[8]	336	0.80						10.62	51	0.21	15.18	3.16	6.77	18	0.38	5.36	2.01	3.00	20	5.95	0.89
TGME49_233480	SRS29C	372	0.79						12.10	49	0.25	13.17	3.25	6.65	17	0.39	4.57	1.79	12.60	84	22.58	3.39
TGME49_245490	MIC8	684	0.84	26.03	31	0.84	4.53	3.81											13.95	93	13.60	2.04
**TGME49_254720**	**GRA8**[9]	269	0.85	76.66	91	0.84	33.83	28.50	65.02	246	0.26	91.45	24.17						25.80	172	63.94	9.59
**TGME49_259020**	**BAG1**[9]	229	0.56	50.72	59	0.86	25.76	22.15	25.81	109	0.24	47.60	11.27	14.51	41	0.35	17.90	6.34	6.00	40	17.47	2.62
TGME49_270240	MAG1[9]	452	0.82						19.96	94	0.21	20.80	4.42						24.45	163	36.06	5.41
**TGME49_270250**	**GRA1**[8]	190	0.80						19.65	91	0.22	47.89	10.34	12.77	35	0.36	18.42	6.72	4.20	28	14.74	2.21
**TGME49_271050**	**SAG2A**[8]	187	0.88						11.61	46	0.25	24.60	6.21	24.98	70	0.36	37.43	13.36	3.75	25	13.37	2.01
TGME49_275440	GRA6	224	0.69	48.46	58	0.84	25.89	21.64	41.53	171	0.24	76.34	18.54	1.38	5	0.28	2.23	0.62	24.60	164	73.21	10.98
TGME49_277720	GDA1/CD39	857	0.76																34.95	233	27.19	4.08
TGME49_280570	SRS35A	172	0.78						15.86	65	0.24	37.79	9.22	19.08	55	0.35	31.98	11.10	4.35	29	16.86	2.53
TGME49_308020	SAG3	385	0.84						13.53	58	0.23	15.06	3.51	2.01	5	0.40	1.30	0.52	8.10	54	14.03	2.1
TGME49_309590	ROP1	446	0.91	102.25	119	0.86	26.68	22.93	44.83	212	0.21	47.53	10.05						50.85	339	76.01	11.4
**TGME49_319560**	**MIC3**[8]	383	0.91						45.04	201	0.22	52.48	11.76	4.33	14	0.31	3.66	1.13	10.20	68	17.75	2.66
TGME49_320170	SRS16E	393	0.91	4.95	6	0.82	1.53	1.26	24.51	102	0.24	25.95	6.24						15.90	106	26.97	4.05

[1] APRANK score from Ricci et al. (2021)

[2] t = algorithm-specific threshold

[3] s = sum of score of 6 or more consecutive aa above algorithm-specific threshold t

[4] c = number of aa contributing to s

[5] s/c = average score per contributing aa

[6] % = number of aa contributing to s in relation to protein length, in % (c*100/ protein length)

[7] cEPS = s*100/protein length

[8] see **A** for plot

[9] antigens included in addition to the APRANK training set

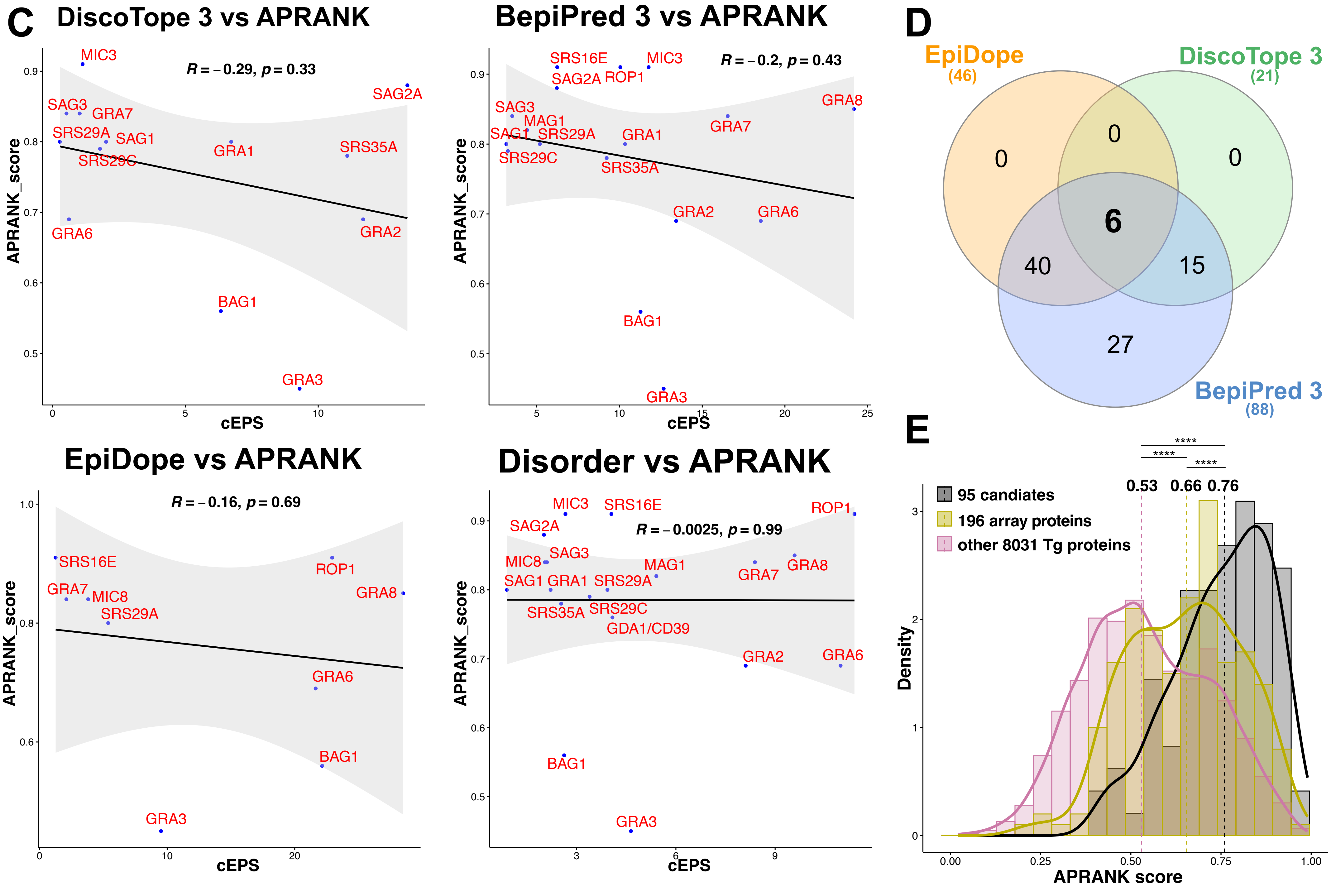
C
DiscoTope 3 vs APRANK
R = −0.29, p = 0.33
MIC3
SAG2A
SAG3
GRA7
SRS29A
SAG1
GRA1
SRS35A
SRS29C
GRA6
GRA2
BAG1
GRA3
APRANK_score
cEPS
BepiPred 3 vs APRANK
R = −0.2, p = 0.43
SRS16E
MIC3
SAG2A
ROP1
GRA8
SAG3
MAG1
GRA7
SAG1
SRS29A
GRA1
SRS29C
SRS35A
GRA2
GRA6
BAG1
GRA3
APRANK_score
cEPS
EpiDope vs APRANK
R = −0.16, p = 0.69
SRS16E
ROP1
GRA7
MIC8
GRA8
SRS29A
GRA6
BAG1
GRA3
APRANK_score
cEPS
Disorder vs APRANK
MIC3
SRS16E
ROP1
SAG2A
R = −0.0025, p = 0.99
SAG3
MIC8
MAG1
GRA7
GRA8
SAG1
GRA1
SRS29A
SRS35A
SRS29C
GDA1/CD39
GRA2
GRA6
BAG1
GRA3
APRANK_score
cEPS
D
EpiDope
(46)
DiscoTope 3
(21)
BepiPred 3
(88)
0
0
0
6
40
15
27
E
95 candiates
196 array proteins
other 8031 Tg proteins
0.53
0.66
0.76
****
Density
APRANK score
