# Supplementary figures and images for: Limited value of current and new in silico predicted oocyst-specific proteins of Toxoplasma gondii for source-attributing serology
Source: Front Parasitol. 2023 Nov 27;2:1292322. doi: 10.3389/fpara.2023.1292322 (PMC11731929; doi:10.3389/fpara.2023.1292322)

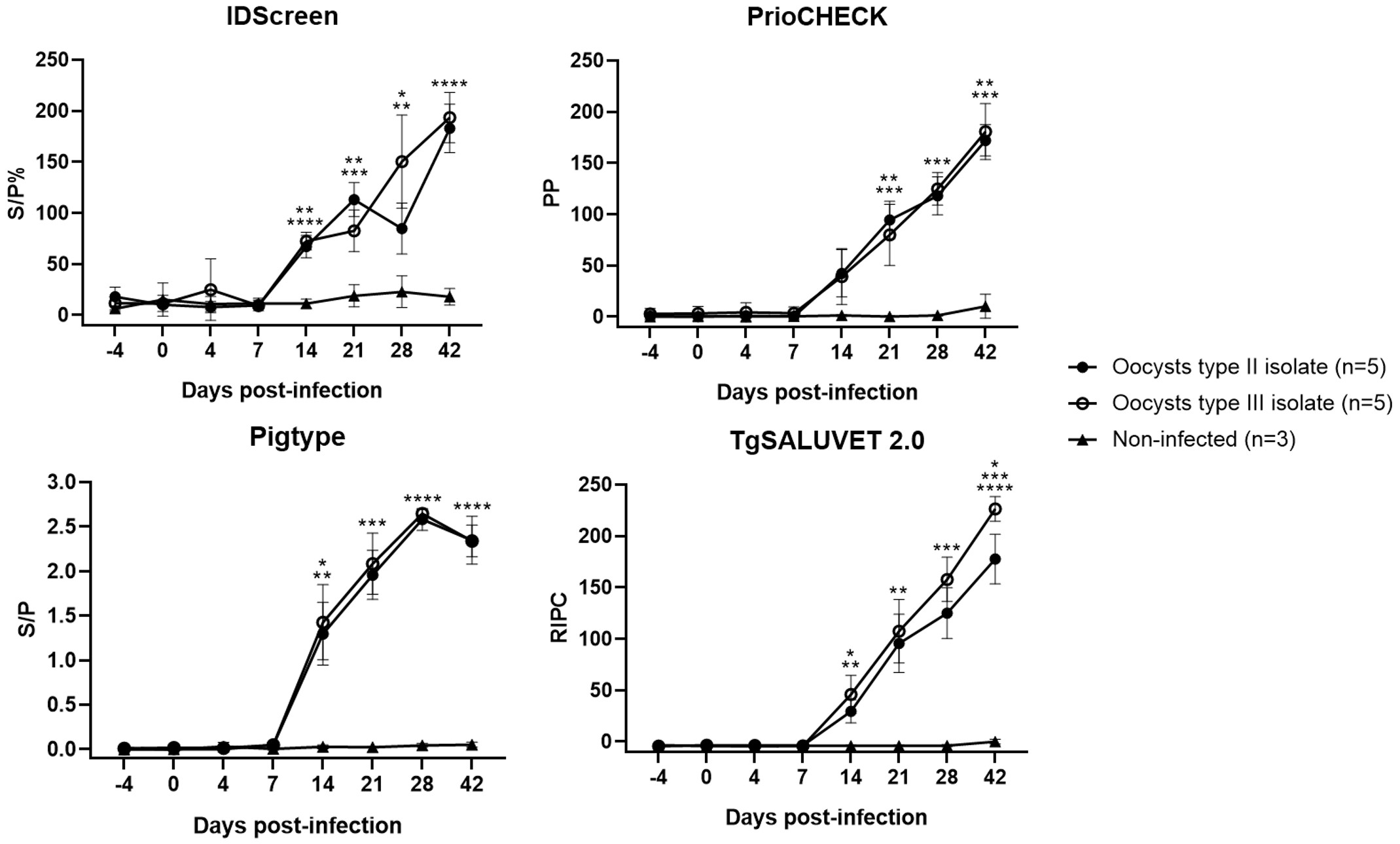

Supplement: Supplementary Figure 2 — Anti-Toxoplasma IgG kinetics in experimentally infected pigs with oocysts from type II and III isolates (panel 2) based on different enzyme-linked immunosorbent assays (IDScreen, PrioCHECK, Pigtype and TgSALUVET 2.0). Seroconversion was recorded from 14-21 days post-infection by all ELISA tests (significance not represented in the figures). Significant differences between experimental groups within sampling days are identified in the figures as follow for each ELISA: *= P< 0.05, **: P< 0.01, ***= P< 0.001 and P< 0.0001. Seroconversion was detected from 14-21 days post-infection and differences between pigs infected with oocysts from type II versus type III isolates were recorded only with TgSALUVET 2.0 at 42 days post-infection. [file Image_2.jpeg]
